# Commentary: Intravenous fentanyl vs. topical lignocaine for ProSeal™ laryngeal mask airway insertion with propofol induction

**DOI:** 10.3389/fmed.2023.1147730

**Published:** 2023-04-18

**Authors:** Sun Zhongpeng, Yang Dong

**Affiliations:** Department of Anesthesiology, Plastic Surgery Hospital of Chinese Academy of Medical Sciences & Peking Union Medical College (CAMS&PUMC), Beijing, China

**Keywords:** supraglottic airway, fentanyl, lignocaine, ProSeal™ laryngeal mask airway, propofol

## Introduction

With the development of the supraglottic airway, the use of a laryngeal mask airway is becoming increasingly widespread for anesthesiologists. Therefore, we should strictly control the indications for the use of a laryngeal mask airway.

In a recent randomized, controlled, double-blind cohort study, Rahmat Ameen Noorazyze et al. (1) discovered that topical spraying of lignocaine was more effective than intravenous fentanyl in inserting the ProSeal™ laryngeal mask airway (PLMA). Aside from the shortcomings mentioned in the article, the authors must clarify several methodological issues.

First, it was generally known that the PLMA is a second-generation reusable inflatable laryngeal mask airway (2). Thus, we would like to know the cuff pressure of the PLMA in this trial and whether it was monitored and adjusted. If not, this may be a potential drawback of the study. Furthermore, determining the optimal timing for PLMA removal was a major challenge for the anesthetist, as it could potentially result in emergency adverse airway events such as laryngospasm. We are interested in understanding whether the patients were fully awake or had an optimal level of anesthesia when the PLMA was removed after the procedure.

Second, while sevoflurane was utilized for intraoperative anesthetic maintenance in this trial, the authors did not specify which opioid was used for perioperative anesthetic maintenance or when it was withdrawn. Reliable data shows that the administration of opioids may minimize airway reactions after mask removal, such as choking, laryngospasm, and other airway-related adverse effects (3). Furthermore, the authors did not specify the type of surgery or the duration of the procedure or anesthesia. These unknown variables could potentially bias the outcomes of their investigation.

Third, the PLMA was inserted without a muscle relaxant, which could potentially induce impairment of the mucosa in the supraglottis. However, an essential sign of injury to the airway mucosa, the presence of blood on the PLMA after removal, was not documented.

Their findings have positive implications for the clinical use of PLMA. If the authors could provide more information and increase the transparency of the study, it may significantly improve its credibility.

## Author contributions

SZ drafted the manuscript. YD carefully read and revised the manuscript. Both authors read and approved the final version of the manuscript.
